# Commentary: The Incidence of Node-Positive Non-small-Cell Lung Cancer Undergoing Sublobar Resection and the Role of Radiation in Its Management

**DOI:** 10.3389/fonc.2020.01558

**Published:** 2020-09-23

**Authors:** Giulia Veronesi

**Affiliations:** ^1^School of Medicine, Vita-Salute San Raffaele University, Milan, Italy; ^2^Division of Thoracic Surgery, IRCCS San Raffaele Scientific Institute, Milan, Italy

**Keywords:** lung cancer, lymph node dissection, thoracic surgery, segmentectomy, radiotherapy

## Introduction

Surgical treatment offers the best chance of cure for early-stage NSCLC (1), but adequate oncological principles are imperative to offer positive long-term outcomes together with reduced surgical trauma. Today, this is possible thanks to modern minimally invasive surgical techniques (2, 3).

The paper by Varlotto et al. (4), through a retrospective analysis of the American National Cancer Database (NCDB), focuses the attention on patients undergoing sublobar resection for non-small cell lung cancer with positive hilar or mediastinal lymph nodes and tries to assess the incidence, risk factors, and prognosis of this particular circumstance, evaluating the potential role of postoperative radiation therapy for these patients.

The topic is of great interest, considering the growing number of patients with smaller and ground-glass early-stage lung cancers that are now treated with segmental resections, partially as a consequence of the greater diffusion of screening investigations in subjects at risk (5, 6) and the use of imaging for other diseases. Indications for sublobar resections, once restricted to patients unsuitable for lobar resection (7), have now been extended to patients without comorbidities (7–9) in light of the good oncological results obtained for patients with small lesions in prospective studies. One essential oncological principle recommends negative hilar or mediastinal nodes at frozen section, and conversion to lobectomy in case this condition is not met (7).

The article by Varlotto et al. (4) is an occasion to reflect on the consequences of a suboptimal situation in which a sublobar resection is undertaken despite the oncological criteria are not followed.

## Inadequate Sublobar Resection

The paper by Varlotto et al. (4) reveals that almost half of the patients undergoing sublobar resection received no removal of a single hilar or mediastinal lymph nodes. This suboptimal staging is likely responsible for the observed reduced long-term oncological outcome of patients undergoing sublobar resections due to an upstaging failure effect. In fact, the authors (4) showed a nodal upstaging rate ranging from 7.6% for nodules under 2 cm to 15% for nodules over 2 cm treated with sublobar resection.

While patients with non-solid lesions have a negligible percentage of metastatic lymph nodes [so that the need of extended lymphadenectomy is discussed (10)], we know that the number of resected lymph nodes is a prognostic factor even in patients treated with sublobar resection for solid tumors smaller than 2 cm, presumably because extended lymph-node dissection avoids mis-staging (11). According to the paper by Yendamuri et al., the extension of lymphadenectomy appears to have a greater impact on survival than the extension of the resection itself (segmentectomy vs. wedge resection). Similarly, Stiles et al. (12) demonstrated that poorer lymph-node dissection in sublobar resection was responsible for the poorer oncologic outcome of these patients, compared to those receiving lobectomy. They did propensity score matching of patients with NSCLC less than 2 cm in size who underwent lobectomy vs. sublobar resection in the SEER database and found that, after matching for an adequate lymph-node dissection, the sublobar resection group no longer had worse survival compared to lobectomy.

It is known that anatomical segmentectomies performed by a minimally invasive approach are technically demanding procedures, with the expected result that much more wedge resections instead of anatomical segments have been done to avoid open surgery (4).

The robotic approach, on the contrary, provides an easier tool to perform an anatomical sublobar resection using a minimally invasive method, improving the quality of the surgery and radicality during lymph-node dissection. The robotic availability of infrared light camera can be of help in reducing the risk of marginal resections, thanks to the better visualization of intersegmental plane with EV ICG injection during the procedure (3, 13).

## Impact of PORT on Overall Survival In Patients Undergoing Sublobar Resection and Positive Lymph Nodes

Importantly, the authors found a protective effect of PORT on the overall survival of patients submitted to SLR with N2 disease when a radical dose higher than 44 Gy was administered. In addition, they found that overall survival (OS) was better for lobectomy compared to sublobar resection, even after matching for different prognostic variables. The value of survival assessment using these large retrospective population databases should be considered with caution, due to the impossibility to adjust for all the preoperative variables that may have influenced the choice of one treatment vs. the other. This selection bias can have an even bigger impact as essential information such as comorbidities, including pulmonary functional data and cardiac comorbidity, fragility index, and ASA score are not available. Thus, no conclusions on OS seem to be reasonable in this condition. The same applies to the role of postoperative treatment, as we do not know the reasons for which one patient received the treatment and another was assigned only for follow-up or chemotherapy. Even in this case, it is possible that the general conditions, complications, or other variables that can have had a negative impact on survival may be the same that hampered the postoperative radiation treatment.

## Preoperative Variables Associated To Lymph-Node Metastases

The authors' objective was to examine the preoperative variables associated with positive nodes to try to better select subjects who should undergo radical lymph-node dissection. They found that some variables were associated with increased lymph-node positivity rate; however, most of them were not related to the disease or clinical status of the patients but to the facility type and site, or type of insurance of the patients, showing that, at the basis of the differences, there could also be a disparity in the access to therapeutic options, with potential inadequate or suboptimal staging and low quality of surgery in subgroups of patients.

## Discussion

The future of the surgical management of lung cancer will depend on the capability of surgeons to offer high-quality, minimally invasive, safe, and radical thoracic procedures tailored to patients and tumor characteristics, to strengthen the value of surgery compared to the emerging role of other non-surgical local treatments such as stereotactic radiotherapy.

In a future perspective, molecular diagnostic and prognostic markers (14) including microhistology tissue based or circulating signatures, in combination with already known prognostic factors, such as volume doubling time, SUV of nodules at CT/PET, and features of nodules at CT scan, hopefully integrated in an AI-based big data analytics platform, will support clinical decisions and guide surgeons on the optimal choice of treatment and extension of surgical resection.

## Author Contributions

GV conceived the idea and wrote the manuscript.

## Conflict of Interest

GV has financial relationship with AB Medica Spa, Johnson & Johnson and Medtronic for consultation and proctoring.
